# Isolated hypercholesterolemia leads to steatosis in the liver without affecting the pancreas

**DOI:** 10.1186/s12944-017-0537-z

**Published:** 2017-07-27

**Authors:** Csaba Csonka, Tamás Baranyai, László Tiszlavicz, Hedvig Fébel, Gergő Szűcs, Zoltán V. Varga, Márta Sárközy, László G. Puskás, Otilia Antal, Andrea Siska, Imre Földesi, Péter Ferdinandy, László Czakó, Tamás Csont

**Affiliations:** 1 Metabolic Diseases and Cell Signaling Research Group, Department of Biochemistry, University of Szeged, Dóm tér 9, Szeged, H-6720 Hungary; 2 1st Department of Internal Medicine, University of Szeged, Szeged, Hungary; 3 Department of Pharmacology and Pharmacotherapy, Semmelweis University, Budapest, Hungary; 4 Department of Pathology, University of Szeged, Szeged, Hungary; 5 Research Institute for Animal Breeding, Nutrition and Meat Science, Herceghalom, Szeged, Hungary; 6 Department of Physiology, Anatomy and Neuroscience, Faculty of Science and Informatics, University of Szeged, Szeged, Hungary; 7 Institute of Genetics, Biological Research Center, Hungarian Academy of Sciences, Szeged, Hungary; 8 Department of Laboratory Medicine, Faculty of Medicine, University of Szeged, Szeged, Hungary; 9 Pharmahungary Group, Szeged, Hungary

**Keywords:** Fatty acid desaturase (FADS), Isolated hypercholesterolemia, Lipidomics, Non-alcoholic fatty liver disease, Stearoyl-coenzyme a desaturase (SCD), Lipotoxicity, Lipid droplets

## Abstract

**Background:**

Lipid accumulation in the liver and pancreas is primarily caused by combined hyperlipidemia. However, the effect of isolated hypercholesterolemia without hypertriglyceridemia is not fully described. Therefore, our aim was to investigate whether hypercholesterolemia alone leads to alterations both in hepatic and pancreatic lipid panel and histology in rats.

**Methods:**

Male Wistar rats were fed with 2% cholesterol +0.25% cholate-supplemented diet or standard chow for 12 weeks. Blood was collected at weeks 0, 4, 8 and 12 to measure serum cholesterol and triglyceride levels. At week 12, both the pancreas and the liver were isolated for further histological and biochemical analysis. Hepatic and plasma fatty acid composition was assessed by gas chromatography. Expression of mRNA of major enzymes involved in saturated/unsaturated fatty acid synthesis was analyzed by qPCR. In separate experiments serum enzyme activities and insulin levels were measured at week 9.

**Results:**

At week 12, rats fed with 2% cholesterol +0.25% cholate-supplemented diet were characterized by elevated serum cholesterol (4.09 ± 0.20 vs. 2.89 ± 0.22 mmol/L, *p < 0.05) while triglyceride (2.27 ± 0.05 vs. 2.03 ± 0.03 mmol/L) and glucose levels (5.32 ± 0.14 vs. 5.23 ± 0.10 mmol/L) remained unchanged. Isolated hypercholesterolemia increased hepatic lipid accumulation, hepatic cholesterol (5.86 ± 0.22 vs. 1.60 ± 0.15 ng/g tissue, *p < 0.05) and triglyceride contents (19.28 ± 1.42 vs. 6.78 ± 0.71 ng/g tissue, *p < 0.05), and hepatic nitrotyrosine level (4.07 ± 0.52 vs. 2.59 ± 0.31 ng/mg protein, *p < 0.05). The histology and tissue lipid content of the pancreas was not affected. Serum total protein level, alanine aminotransferase (ALT) and aspartate aminotransferase (AST) activities remained unchanged in response to isolated hypercholesterolemia while serum alkaline phosphatase activity (ALP) significantly increased. Plasma insulin levels did not change in response to isolated hypercholesterolemia suggesting an intact endocrine function of the pancreas. Isolated hypercholesterolemia caused a significantly increased hepatic and serum fatty acid level associated with a marked alteration of fatty acid composition. Hepatic expression of Δ9-desaturase (SCD1) was increased 4.92×, while expression of Δ5-desaturase and Δ6-desaturase were decreased (0.447× and 0.577×, respectively) due to isolated hypercholesterolemia.

**Conclusions:**

Isolated hypercholesterolemia leads to hepatic steatosis and marked alterations in the hepatic lipid profile without affecting the pancreas. Altered fatty acid profile might mediate harmful effects of cholesterol in the liver.

## Background

Hyperlipidemia has been shown to affect morphology, metabolism, and biological functions of several organs including e.g. the liver [1, 2], pancreas [2–4], heart [5–9], vasculature [10], skeletal muscle [11], etc. Based on the key lipid component [i.e. triglyceride and/or cholesterol], hyperlipidemia could be classified into three types: (i) isolated hypertriglyceridemia, (ii) isolated hypercholesterolemia, and (iii) combined hyperlipidemia (both hypertriglyceridemia and hypercholesterolemia) [12].

In Western societies, combined hyperlipidemia could be developed as a consequence of sedentary lifestyle, dietary habits and obesity. Combined hyperlipidemia is a common disease in the population aged 20 and over. Its prevalence was 13.4% in the United States in 2009–2010 [13]. According to a Mexican survey, the simultaneous elevation of cholesterol and triglyceride concentrations was observed in 12.6% of the general population (cholesterol concentration above 6.3 mmol/L [240 mg/dl]) [14]. Nevertheless, in this study, 3.5% of all individuals was found to have isolated hypercholesterolemia [14].

It is well-established that clinically manifested combined hyperlipidemia could induce severe fatty degeneration in the hepatocytes termed non-alcoholic fatty liver disease [15]. It may develop into non-alcoholic steatohepatitis in 20–30% of the cases [16] and potentially lead to hepatic fibrosis [17]. Combined hyperlipidemia was found to induce acute pancreatitis with characteristic histological alterations [18–20]. Furthermore, acute pancreatitis induced by cerulein, taurocholate or L-arginine was suggested to be aggravated in the presence of combined hyperlipidemia [21, 22]. Other studies have mainly focused on the fatty infiltration of the pancreas in response to hyperlipidemia and obesity [23], however, these mostly epidemiological/pathological reports did not indicate the type of hyperlipidemia. Nevertheless, experimental combined hyperlipidemia has been shown to cause increased pancreatic triglyceride accumulation in rats and mice as assessed by both biochemical and histological tools [20, 24].

Interestingly, isolated hypercholesterolemia is less surveyed in clinical studies. Therefore, mainly experimental data demonstrated deterioration of normal liver architecture, lipid infiltration and mild liver fibrosis in rats in response to isolated hypercholesterolemia [25–27]. However, to the best of our knowledge no study has been performed to elucidate the effects of isolated hypercholesterolemia on pancreatic tissue.

The majority of studies investigating the accumulation of lipids in different organs have been focused either on the liver or on the pancreas. In case of epidemiological reports, the type of hyperlipidemia is not always clear. Moreover, relatively few studies have examined the effects of certain types of hyperlipidemia both on the liver and the pancreas in the same experimental setup. It has been reported that combined hyperlipidemia caused an increased amount of intracellular lipid droplets in the liver, whereas the pancreas remained unchanged under the same conditions [28, 29]. In contrast, both pancreatic and hepatic triglyceride contents were higher in mice due to combined hyperlipidemia in other studies [24, 30]. Nonetheless, the effects of isolated hypercholesterolemia on the lipid content of the liver and the pancreas have not been investigated in the same experimental setup.

Therefore, the aim of our study was to investigate whether isolated hypercholesterolemia leads to simultaneous development of steatosis in the liver and the pancreas. Therefore, we investigated the morphology and major lipid panel (cholesterol and triglycerides) both of the liver and the pancreas in a non-obese rat model of isolated hypercholesterolemia (2% cholesterol +0.25% cholate-supplemented diet for 12 weeks without any extra triglyceride administration).

## Methods

### Animals and experimental protocol

The investigation conforms to the Guide for the Care and Use of Laboratory Animals published by the U.S. National Institutes of Health [National Institutes of Health publication 85–23, revised 1996], and was approved by the local animal ethics committee of the University of Szeged.

**Fig. 1:**
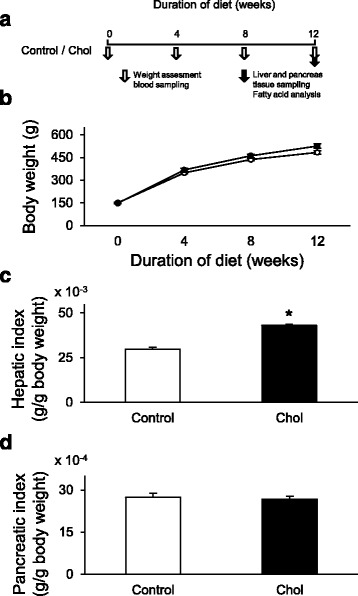
**a** Experimental protocol; **b** Body weight of control (o) and isolated hypercholesterolemic (●) rats in week 0, 4, 8, and 12; **c** liver weight index (liver weight/body weight) and **d** pancreas weight index (pancreas weight/body weight) at the end of the diet. Data are mean ± SEM, *n* = 12, respectively, *p < 0.05 vs. control

In order to induce isolated hypercholesterolemia, male Wistar rats (130–164 g) were fed with laboratory chow supplemented with 2% (*w*/w) cholesterol (Hungaropharma Zrt., Budapest, Hungary) and 0.25% (*w*/w) sodium-cholate-hydrate (Sigma, St. Louis, MO) for 12 weeks (Fig. 1a) as described previously [31, 32]. Control rats were fed with standard rat chow. Body weight and serum cholesterol, triglyceride, and blood glucose levels were measured at weeks 0, 4, 8, and 12 (Fig. 1a). At the end of the diet period, the liver and the pancreas were isolated from both groups in order to perform histological analysis and to measure tissue cholesterol and triglyceride levels. In addition, hepatic nitro-oxidative stress was assessed by measuring tissue 3-nitrotyrosine level, and characterization of fatty acid composition of the liver and plasma was performed by gas chromatography.

### Measurement of serum lipid and blood glucose levels

Blood samples were collected from the saphenous vein after overnight fasting. Blood glucose level was immediately determined by a standard glucose meter in duplicates (Accutrend GCT, Roche Diagnostics, Mannheim, Germany) as described previously [33–36].

For serum lipid measurements, collected blood was allowed to clot and then was centrifuged (2000 g, 15 min, 4 °C). Serum cholesterol and triglyceride levels were assayed by enzymatic colorimetric assays (Cholesterol PAP and Triglyceride PAP assays; Diagnosticum, Budapest, Hungary) as described earlier [37, 38].

### Measurement of tissue lipid levels

Liver and pancreas samples were homogenized (homogenization buffer: 20 mM TrisBase; 150 mM NaCl; pH 7.8; 0.05% TritonX100) with a sonicator (4 °C; 3 × 10 s; Hielscher UP100H Ultrasonic Processor [Hielscher Ultrasonics, Teltow, Germany]) and centrifuged (1000 g; 5 min; 4 °C). Tissue cholesterol and triglyceride were measured from the supernatant by enzymatic colorimetric assays according to the manufacturer’s instructions (CHOD-PAP and GPO-PAP assays; Cobas-Roche, West Sussex, England).

### Measurement of serum enzyme activities and plasma insulin levels

In separate experiments blood samples were collected from the aorta after overnight fasting in a group of male Wistar rats fed with normal or 2% (*w*/w) cholesterol and 0.25% (*w*/w) sodium cholate hydrate supplemented standard chow for 9 weeks.

Serum liver enzyme activities including alanine aminotransferase (ALT), aspartate aminotransferase (AST) and alkaline phosphatase (ALP) as well as plasma total protein concentration were measured to monitor the effect of cholesterol and cholic acid enriched diet on liver function. Serum ALT, AST and ALP activities were measured with test kits from Roche Diagnostics (Mannheim, Germany) on Roche Modular P800 chemistry analyzer. Plasma total protein concentration were determined by a bicinchoninic acid protein assay kit (Pierce, BCA protein assay kit) in triplicates according to the manufacturer’s instructions.

Serum pancreatic enzyme activities including amylase and lipase were measured to determine the effect of cholesterol plus cholic acid enriched diet on the exocrine pancreas function. Serum amylase (EPS-G7 substrate) and lipase activities were measured with test kits from Diagnosticum (Budapest, Hungary) and Roche Diagnostics (Mannheim, Germany), respectively on Roche Modular P800 chemistry analyzer.

To monitor the effect of cholesterol and cholic acid enriched diet on the endocrine pancreas, plasma insulin levels were measured by an enzyme immunoassay (Mercodia, Ultrasensitive Rat Insulin ELISA) in duplicates according to the manufacturer’s instructions as described previously [33–36].

### Histology

Samples from the liver and the pancreas tail were fixed overnight in 4% (*v*/v) neutral formaldehyde solution and embedded in paraffin. Four μm tissue slices were subjected to hematoxylin-eosin (liver, pancreas), toluidine blue (liver), and Crossman’s trichrome (liver) staining. The histology was scored by the method described previously [39, 40] with minor modifications by an expert pathologist in a blinded manner. In case of the liver, 6 different signs: (i) xanthomatous alterations, (ii) steatotic microvesicles, (iii) portal fibrosis, (iv) hyperplasia of Kuppfer cells, (v) biliary cell proliferation and (vi) lobular inflammation; in case of the pancreas 5 different signs: (i) vacuolization, (ii) fatty infiltration, (iii) relative number of islets, (iv) islet deformations and (v) hemosiderin content were graded from 0 to 3, respectively. In this scale 0 = absent, 1 = mild, 2 = moderate and 3 = severe representation of histological signs. The total alteration was calculated by summarizing the scores for the different parameters.

### Fatty acid analysis

Plasma samples (2.5–4 mL) were saponified using 2.5 mL of 18.6 mol/L NaOH and 40 mL of methanol. Nonadecanoic acid (0.7 mg) was used as an internal standard (1 g/L in chloroform/isopropanol (1:2, *v*/v). The solution was heated in 80 °C for 50 min, and samples were acidified with citric acid, extracted with chloroform and methylated with methanol and methanolic-boron-trifluoride. Liver samples (1200 mg) were prepared as described above by using 5 mg of internal standard. Fatty acid methyl esters were separated by gas-liquid chromatography on a Shimadzu 2010 chromatograph (Shimadzu Corporation, Tokyo, Japan) fitted with automatic sampler AOC-20 and FID detector. The used column was a CP Sil 88 fused capillary column (50 m × 0.25 mm × 0.20 μm film thickness). Helium was used as carrier and make-up gas. The split ratio was 50:1. The injection port temperature was set to 270 °C and the detector was 300 °C. Oven temperature was increased from 80 °C to 205 °C by 1.7 °C/min, held for 5 min then increased to 225 °C by 10 °C/min, held for 20 min. Individual fatty acids were identified by comparison of gas chromatography peaks with peaks of known standards (Mixture Me 100, Larodan Fine Chemicals AB, Limhamn, Sweden). Fatty acid composition was expressed as μg fatty acid per g liver or plasma wet weight.

### Measurement of hepatic 3-nitrotyrosine level

To further characterize hepatic damage induced by isolated hypercholesterolemia 3-nitrotyrosine level was measured in liver samples as a marker of tissue nitro-oxidative stress using ELISA (Cayman Chemical, Ann Arbor, MI, USA) as described previously [38, 41, 42]. Briefly, homogenates were incubated overnight with nitrotyrosine acetylcholinesterase tracer and anti-nitrotyrosine rabbit IgG in microplates pre-coated with mouse anti-rabbit IgG. Ellman’s reagent was used for development.

### Total RNA isolation and quantitative polymerase chain reaction [qPCR]

Total RNA was isolated from 22 to 30 mg homogenized tissue with RNeasy Fibrous Tissue Mini kit (QIAGEN, Austin, Texas, USA) as described by the manufacturer. DNA was degraded with TURBO™ DNase (Ambion, Life Technologies, Carlsbad, California, USA). RNA concentration was measured with NanoDrop1000 Version 3.8.1. (Thermo Fisher Scientific Inc., Waltham, MA, USA). cDNA synthesis was performed with High Capacity cDNA Reverse Transcription Kit (Applied Biosystems Foster City, CA, USA) and cDNA was diluted 4× for PCR.

qPCR reaction was performed with Rotor-Gene Version 6.0 (Corbett Research, Sydney, Australia). Mastermix was prepared from 5 μL FastStart Essential DNA Green Master (Roche Applied Science, Mannheim, Germany) and 0.5 μL primer solution (10 pM) for one sample and 4.5 μL cDNA was added. The following PCR protocol was applied: heat-start at 95 °C for 10 min; 45 cycles of 95 °C for 15 s; 60 °C for 10 s and 72 °C for 20 s. Tm values were compared and non-template controls were run to verify the specificity of the primers. The sequences of the primers are presented in Table 1. Gene expression was normalized to HPRT1 and PPIA.

**Table 1:** Sequence of the primers utilized in evaluation of fatty acid biosynthesis in livers of rats with isolated hypercholesterolemia

NCBI Reference Sequence	Symbol	Official full name		Sequence
NM_139192.2	SCD1	Stearoyl-coenzyme A desaturase 1 [delta-9-desaturase]	Fw	tgctctgagctgttttgttga
			Rv	cgaaggcatttccagagg
NM_031841.1	SCD2	Stearoyl-coenzyme A desaturase 2 [delta-9-desaturase]	Fw	ggtgtcgatgggagctgt
			Rv	ttgatgtgccagcggtact
NM_053445.2 Transcript variant X1: XM_006231075.2	FADS1	Fatty acid desaturase 1	Fw	gagggcattcatgcacaga
			Rv	aggcagacatggtcacacc
NM_031344.2 Transcript variant X1: XM_008760244.1	FADS2	Fatty acid desaturase 2	Fw	agagaagccgctgctgag
			Rv	tgcttcatttgtggaggtagg
NM_017332.1	FASN	Fatty acid synthase	Fw	ttcagagctacagaaggtgctaga
			Rv	tctaactggaagtgacggaagg
NM_012583.2	HPRT1	Hypoxanthine phosphoribosyltransferase 1	Fw	gaccggttctgtcatgtcg
			Rv	acctggttcatcatcactaatcac
NM_017101.1	PPIA	Peptidylprolyl isomerase A [cyclophilin A]	Fw	tctgcactgccaagactgag
			Rv	catgccttctttcaccttcc

### Statistical analysis

Data were expressed as means ± standard error of the mean (SEM). Serum enzyme activities (ALT, AST, ALP, amylase, lipase) and plasma total protein as well as insulin levels, liver and pancreas cholesterol and triglyceride content, hepatic nitro-tyrosine as well as individual fatty acid contents were analyzed with Student *t*-test. Body weight, serum cholesterol and triglyceride levels were analyzed by repeated measures ANOVA followed by Dunn’s post hoc test. Histological scores were analyzed by Mann-Whitney U test. qPCR results were evaluated by Welch’s test. *P* < 0.05 was accepted as statistically significant difference.

## Results

### Animal and organ weights

Body weight was not affected significantly in the hypercholesterolemic group compared to the control group (Fig. 1b). In order to compare individual cases properly, organ weights were indexed by body weights. Liver weight index significantly increased in the hypercholesterolemic group (Fig. 1c); however, there was no significant difference in terms of pancreas index between control and hypercholesterolemic groups (Fig. 1d).

### Serum cholesterol, triglyceride and blood glucose

**Fig. 2:**
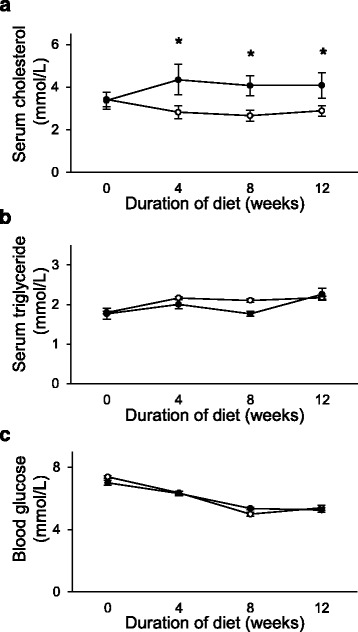
**a** Serum Cholesterol (*n* = 6–9); **b** serum triglyceride (*n* = 7–9); and **c** serum blood glucose (*n* = 6–9) in control (o) and isolated hypercholesterolemic (●) rats in week 0, 4, 8, and 12. Data are mean ± SEM, *p < 0.05 vs. control

To validate the development of isolated hypercholesterolemia, cholesterol and triglyceride concentrations were measured in the serum of the animals. Elevation of serum cholesterol concentration was observed due to cholesterol-supplemented diet on weeks 4, 8 and 12 (Fig. 2a), while serum triglyceride concentrations did not change in parallel (Fig. 2b). Blood glucose levels were not different in the hypercholesterolemic animals as compared to the control animals (Fig. 2c).

### Lipid content of the liver and the pancreas

**Fig. 3:**
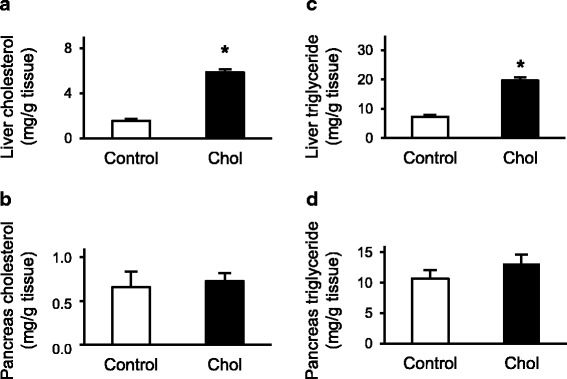
Effect of isolated hypercholesterolemia on **a** pancreas tissue cholesterol, **b** liver tissue cholesterol; **c** pancreas tissue triglyceride; **d** liver tissue triglyceride at the end of the diet. Data are mean ± SEM *n* = 10, respectively, *p < 0.05 vs. control

To analyze the effect of isolated hypercholesterolemia on tissue lipid content, hepatic and pancreatic cholesterol and triglyceride content were assessed at the end of the 12-weeks diet period. Hepatic cholesterol level was significantly elevated in the hypercholesterolemic rats (Fig. 3a), while pancreatic cholesterol level remained unaffected (Fig. 3b). Similarly, hepatic triglyceride content was significantly increased (Fig. 3c), but pancreatic triglyceride content did not change in response to isolated hypercholesterolemia (Fig. 3d).

### Serum enzyme activities and plasma insulin concentration

**Fig. 4:**
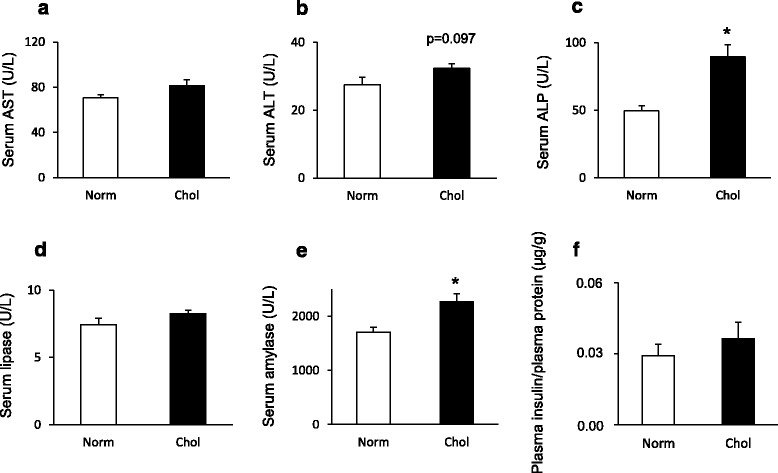
Effect of isolated hypercholesterolemia on serum enzyme activities including **a** AST, **b** ALT, **c** ALP, **d** amylase, **e** lipase and **f** plasma insulin concentration at week 9. Data are mean ± SEM *n* = 7–8, respectively, *p < 0.05 vs. control

Isolated hypercholesterolemia significantly increased serum ALP (Fig. 4c), while only induced a slight but not significant increase in ALT and ASP activities (Fig. 4a and b). Plasma total protein level did not change significantly in response to isolated hypercholesterolemia as compared to the normal group (57.08 ± 1.58 vs. 51.41 ± 2.58 g/L, respectively). To test the effect of isolated hypercholesterolemia on the exocrine and endocrine pancreas function, serum lipase and amylase activity as well as plasma insulin concentrations were determined (Fig. 4d-f). Plasma amylase activity (a non-specific marker for pancreas damage) showed significant increase in the isolated hypercholesterolemic group (Fig. 4e), while lipase activity (a specific marker for pancreas damage) did not change. Plasma insulin concentrations were not different between the two groups (Fig. 4f).

### Histological analyses of the pancreas and the liver

**Fig. 5:**
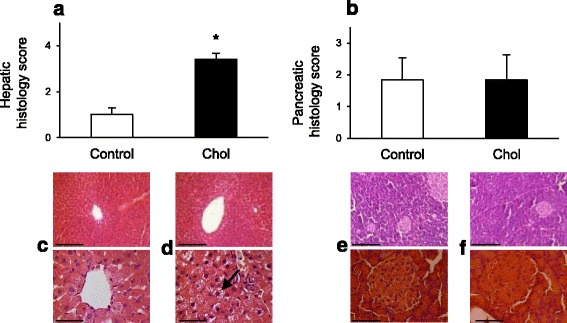
Effect of isolated hypercholesterolemia on hepatic (**a**, *n* = 12) and pancreatic (**b**, *n* = 6) histology evaluated by a score system at the end of the diet. Data are mean ± SEM, *p < 0.05 vs. control. Representative images of histological sections of the liver (**c**: Control, **d**: Chol, scale bars represent 400 μm (*top*) or 100 μm (*bottom*) and pancreas (**e**: Control, **f**: Chol, scale bars represent 200 μm (*top*) or 50 μm (*bottom*) stained with hematoxylin and eosin. *Arrow* shows intracellular lipid droplets in the liver

Isolated hypercholesterolemia significantly altered the histological picture of the liver according to hepatic histology score (Fig. 5a, c, and d; Table 2). Changes were predominantly manifested in xanthomatous alterations and steatotic microvesicles. In contrast, pancreatic histology was not modified by isolated hypercholesterolemia in terms of gross morphological parameters as compared with rats on control diet (Fig. 5b, e, and f; Table 2).

**Table 2:** Histological scores of the liver and pancreas of rats with isolated hypercholesterolemia

LIVER	Xanthomatous alteration	Steatotic microvesicles	Portal fibrosis	Kuppfer hyperplasia	Biliary proliferation	Lobular inflammation
Control	0.00 ± 0.00	0.25 ± 0.13	0.08 ± 0.08	0.17 ± 0.11	0.17 ± 0.11	0.33 ± 0.19
Chol	2.17 ± 0.24	1.08 ± 0.08	0.00 ± 0.00	0.00 ± 0.00	0.08 ± 0.08	0.08 ± 0.08
Significance	*p* < 0.05	*p* < 0.05	n.s.	n.s.	n.s.	n.s.
PANCREAS	Vacuolisation	Fatty infiltration	Relative number of islets	Islet deformations	Hemosiderin content of islets	
Control	0.00 ± 0.00	0.00 ± 0.00	0.83 ± 0.40	1.00 ± 0.37	0.00 ± 0.00	
Chol	0.17 ± 0.17	0.00 ± 0.00	0.67 ± 0.33	1.00 ± 0.52	0.00 ± 0.00	
Significance	n.s.	n.s.	n.s.	n.s.	n.s.	

Control: standard diet; Chol: 2% cholesterol and 0.25% cholic acid enriched diet for 12 weeks; *n* = 6–12

### Nitro-oxidative stress in the liver

**Fig. 6:**
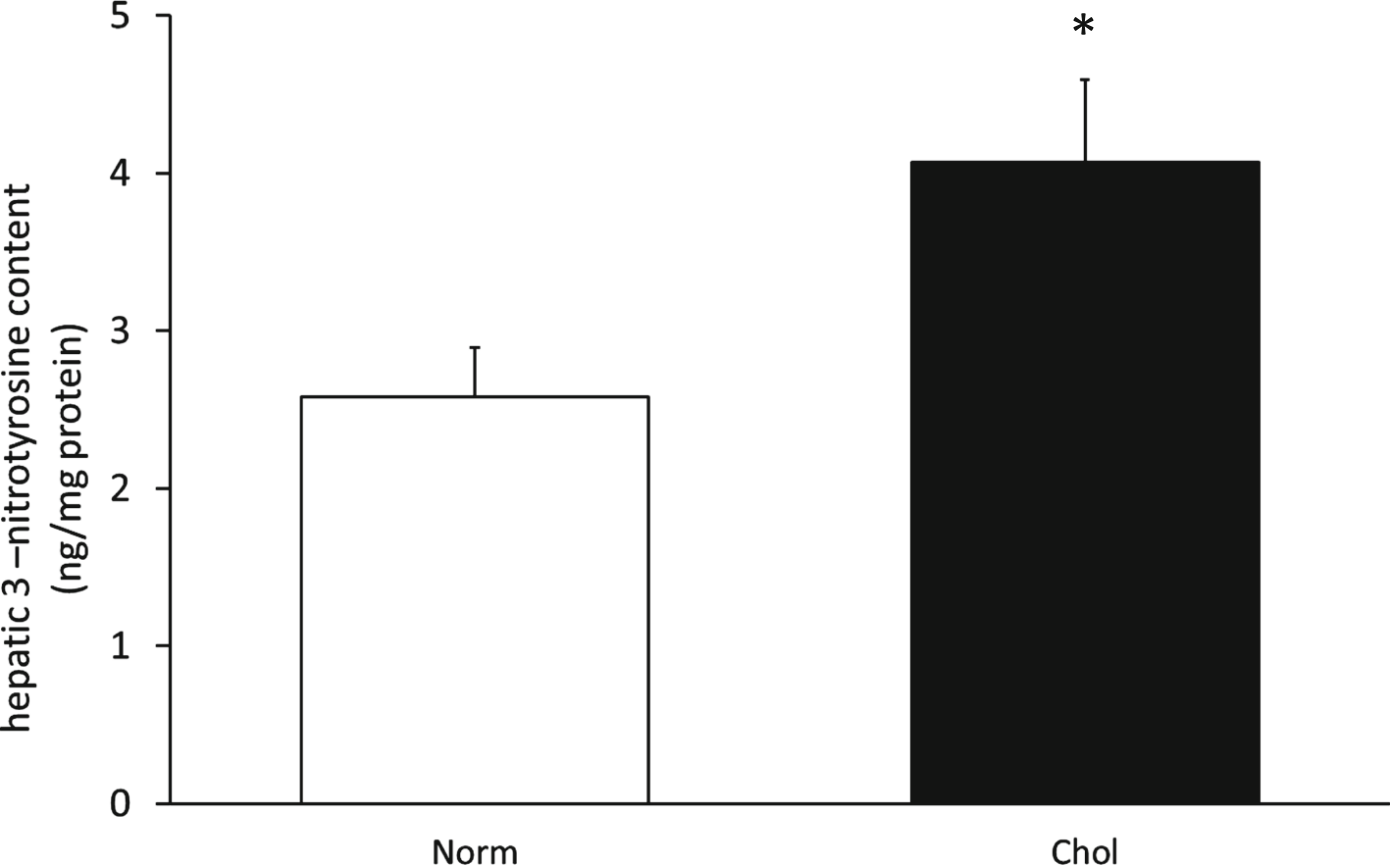
Effect of isolated hypercholesterolemia on hepatic 3-nitrotyrosine level. Data are mean ± SEM, *n* = 8, *p < 0.05 was accepted significant change vs. control (*p* < 0.05)

As we have not experienced any signs of pathological alterations in the pancreas, in the rest of the study we have only focused on hepatic characterization. Since chronic hyperlipidemia induces nitro-oxidative stress in various organs, we have tested hepatic 3-nitrotyrosine levels. We have found that 3-nitrotyrosine was increased significantly due to isolated hypercholesterolemia (Fig. 6).

### Fatty acid composition of the liver and the plasma

In order to further characterize the effects of isolated hypercholesterolemia on tissue and serum lipid profile, we have analyzed the fatty acid composition of the liver and the plasma. Gas chromatographic analysis confirmed that total hepatic fatty acid content was significantly increased due to isolated hypercholesterolemia. In the liver of control animals, the predominant fatty acids were arachidonic (C20:4n-6), linoleic (C18:2n-6), palmitic (C16:0), stearic (C18:0), oleic (C18:1n-9) and docosahexaenoic acid (C22:6n-3, Table 3). In addition to the previously listed fatty acids, palmitoleic acid (C16:1n-7) also became a major component in the liver of hypercholesterolemic rats.

**Table 3:** Fatty acid content of the liver in isolated hypercholesterolemia

		Control		Chol				
Code	Name	Average	SEM	Average	SEM	Fold change	*p* value	
C14:0	Myristic acid	85.0	4.85	476	35.7	5.60	2.68E-10	↑↑
C15:0	Pentadecanoic acid	62.3	7.51	297	20.1	4.77	2.18E-10	↑
C16:0	Palmitic acid	5817	146	12,437	568	2.14	1.28E-10	↑
C16:1n-7	Palmitoleic acid	333	34.5	6768	695	20.28	5.00E-09	↑↑↑
C17:0	Heptadecanoic acid	159	5.65	283	12.5	1.78	6.45E-09	↑
C18:0	Stearic acid	4598	71.1	3218	65.1	0.70	1.25E-12	↓
C18:1n-9	Oleic acid	3306	170	23,114	1385	6.99	1.50E-12	↑↑
C18:2n-6	Linoleic acid	6246	353	18,903	776	3.03	6.10E-13	↑
C18:3n-6	γ-Linolenic acid	75.8	7.63	201	21.5	2.66	1.46E-05	↑
C18:3n-3	α-Linolenic acid	129	12.9	728	44.6	5.64	9.80E-12	↑↑
C20:2n-6	Eicosadienoic acid	111	5.69	442	26.0	3.98	1.88E-11	↑
C20:3n-3	Eicosatrienoic acid	118	11.8	777	52.2	6.55	2.39E-11	↑↑
C20:4n-6	Arachidonic acid	6895	243	6143	130	0.89	0.0125	↓
C20:5n-3	Eicosapentaenoic acid	73.0	6.97	197	10.7	2.71	1.85E-09	↑
C22:5n-3	Docosapentaenoic acid	283	12.6	769	53.5	2.72	1.07E-08	↑
C22:6n-3	Docosahexaenoic acid	1313	50.4	1452	106	1.11	0.2474	n.s.
Total fatty acid content		29,612	941	76,293	3386	2.58	5.55E-12	

Control: standard diet; Chol: 2% cholesterol and 0.25% cholic acid enriched diet for 12 weeks. Fatty acid content is given in μg/g wet weight. *n* = 12

Absolute amount of almost every measured fatty acid was significantly increased in the liver due to isolated hypercholesterolemia most significantly: palmitoleic acid (C16:1n-7), oleic acid (C18:1n-9), eicosatrienic acid (C20:3n-3), α-linolenic acid (C18:3n-3) and myristic acid (C14:0). Nevertheless, the quantity of docosahexaenoic acid (C22:6n-3) did not change significantly and stearic acid (C18:0) and arachidonic acid (C20:4n-6) were significantly decreased in the liver (Table 3).

In the plasma of control and hypercholesterolemic rats the dominant fatty acids were the same as in the liver (Table 4). The pattern of fold-change of individual fatty acids due to cholesterol-enriched diet in the plasma was also similar to changes seen in the liver; however, the degree was less pronounced.

**Table 4:** Fatty acid content of the plasma in isolated hypercholesterolemia

		Control		Chol				
Code	Name	Average	SEM	Average	SEM	Fold change	*p* value	
C14:0	Myristic acid	9.81	0.73	14.0	1.06	1.43	0.0036	↑
C15:0	Pentadecanoic acid	7.69	0.49	14.0	1.18	1.82	0.0001	↑
C16:0	Palmitic acid	455	27.9	521	35.4	1.15	0.1563	n.s.
C16:1n-7	Palmitoleic acid	35.7	5.80	195	29.6	5.46	2.68E-05	↑↑
C17:0	Heptadecanoic acid	9.55	0.60	14.2	1.22	1.49	0.0024	↑
C18:0	Stearic acid	199	9.56	148	11.3	0.74	0.0024	↓
C18:1n-9	Oleic acid	305	27.3	707	69.6	2.32	2.05E-05	↑
C18:2n-6	Linoleic acid	546	37.1	742	49.0	1.36	0.0041	↑
C18:3n-6	γ-Linolenic acid	8.27	0.81	9.05	1.54	1.09	0.6588	n.s.
C18:3n-3	α-Linolenic acid	13.5	1.30	23.9	1.79	1.77	0.0001	↑
C20:2n-6	Eicosadienoic acid	5.35	0.42	12.2	0.77	2.29	9.11E-08	↑
C20:3n-3	Eicosatrienoic acid	6.25	0.84	26.0	2.02	4.16	7.31E-09	↑↑
C20:4n-6	Arachidonic acid	607	31.2	477	35.6	0.78	0.0114	↓
C20:5n-3	Eicosapentaenoic acid	12.4	1.24	11.5	1.28	0.93	0.6432	n.s.
C22:5n-3	Docosapentaenoic acid	16.5	1.35	18.6	1.66	1.13	0.3388	n.s.
C22:6n-3	Docosahexaenoic acid	61.1	3.70	59.9	6.55	0.98	0.8724	n.s.
Total fatty acid content		2304	130	3007	233	1.30	0.0153	

Control: standard diet; Chol: 2% cholesterol and 0.25% cholic acid enriched diet for 12 weeks. Fatty acid content is given in μg/g plasma. *n* = 11–12

### mRNA expression of enzymes involved in fatty acid synthesis

**Fig. 7:**
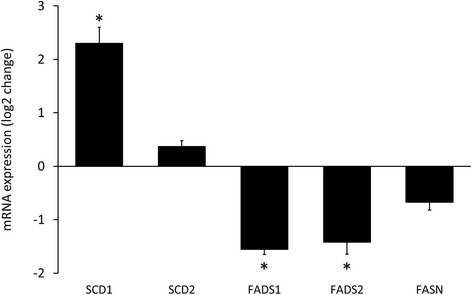
Effect of isolated hypercholesterolemia on mRNA expression of major enzymes involved in the synthesis of saturated and unsaturated fatty acids. Data are mean ± SEM, *n* = 8, *was accepted significant change vs. control (*p* < 0.01). FASN: fatty acid synthase, SCD: stearoyl-coenzyme A desaturase (Δ9-desaturase), FADS1: fatty acid desaturase 1 (Δ5-desaturase), FADS2: fatty acid desaturase 2 (Δ6-desaturase)

**Fig. 8:**
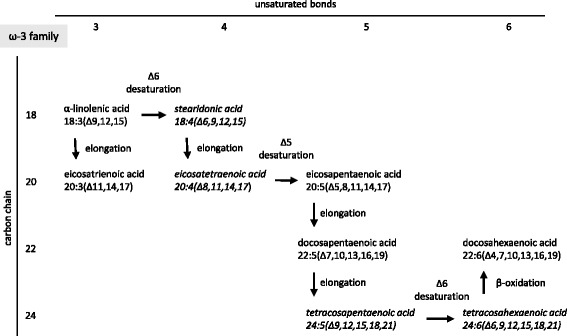
Synthesis of the n-3 (ω-3) fatty acid family members

**Fig. 9:**
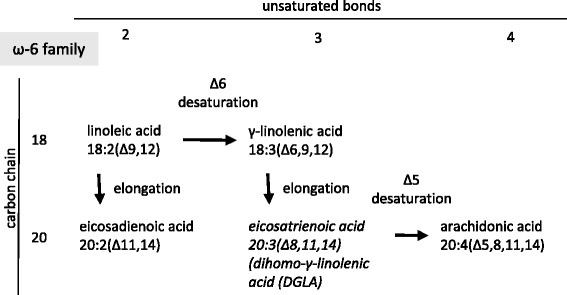
Synthesis of the n-6 (ω-6) fatty acid family members

We investigated the expression of mRNAs of major enzymes involved in the synthesis of saturated and unsaturated fatty acids (Figs. 7, 8 and 9), including fatty acid synthase (FASN), stearoyl-coenzyme A desaturase 1 (Δ9-desaturase; SCD1), stearoyl-coenzyme A desaturase 2 (Δ9-desaturase; SCD2), fatty acid desaturase 1 (Δ5-desaturase; FADS1) and fatty acid desaturase 2 (Δ6-desaturase; FADS2). The expression of mRNAs of FADS1 (Δ5-desaturase) and FADS2 (Δ6-desaturase) significantly decreased in the liver due to isolated hypercholesterolemia. In contrast, the expression of mRNA of stearoyl-coenzyme A desaturase 1 (Δ9-desaturase, SCD1) significantly increased in the liver of hypercholesterolemic rats. However, the levels of FASN and SCD2 mRNAs did not change significantly (Fig. 7).

## Discussion

In the present, study we have investigated the effects of isolated hypercholesterolemia both on hepatic and pancreatic lipid profile and histology. Here we have demonstrated for the first time that a cholesterol-enriched diet leading to the development of isolated hypercholesterolemia resulted in accumulation of not only cholesterol but, interestingly, also triglyceride in the liver. Moreover, increased nitro-oxidative stress and histological signs of hepatic steatosis have also been developed in isolated hypercholesterolemia. However, isolated hypercholesterolemia did not cause any alterations in lipid levels or gross histology in the pancreas in the same experimental setup. In addition, we have found marked alterations in the fatty acid composition of the liver as well as the plasma in hypercholesterolemic rats due to changes in the hepatic expression of SCD1, FADS1 and FADS2.

In developed countries, isolated hypercholesterolemia may constitute a significant proportion of hyperlipidemia with elevated cholesterol level in the adult population [1, 6]. In experimental studies, the development of hyperlipidemia is influenced by a variety of factors including e.g. the type of animals, diet duration, ingredients and their proportion, etc. In our present study, we used a non-obese model of isolated hypercholesterolemia characterized previously by our [31, 32] and other research groups as well [18, 43]. In agreement with these aforementioned studies, we have confirmed here that 2% cholesterol and 0.25% sodium-cholate-hydrate enriched diet for 12 weeks leads to increased serum cholesterol level, without affecting serum triglyceride level in rats.

The major goal of our present study was to compare the effects of isolated hypercholesterolemia both on lipid content and histology of the liver and the pancreas in the same experimental setup. Interestingly, the majority of studies regarding the effects of hyperlipidemia have been focused on either the liver or the pancreas. Thus, in the present study we have demonstrated for the first time in the literature that isolated hypercholesterolemia causes marked cholesterol and also triglyceride accumulation in the liver accompanied by steatotic degeneration, while the pancreas remains unaffected in the same experimental setup. As there was no comparative study conducted, we compared our results only with studies looking at the effect of isolated hypercholesterolemia either on the liver or on the pancreas.

Regarding the effects of isolated hypercholesterolemia on the liver, our findings are in accordance with those of Wang et al. [44], who observed that isolated hypercholesterolemia [induced by a diet supplemented with 2% cholesterol and 0.5% cholic acid] increased liver weight and also induced fatty degeneration of the liver in Wistar rats. In our present study, isolated hypercholesterolemia lead to slightly increased ALT and ASP activities and induced a significant elevation of serum ALP activity. Plasma total protein level did not change in response to isolated hypercholesterolemia as compared to the normal group. These results suggest that isolated hypercholesterolemia leads to steatosis without severely affecting liver function. Some other research groups using different diet supplementation regimes have shown a more severe form of hepatic injury, i.e. hepatic fibrosis due to isolated hypercholesterolemia induced by a diet containing 2% cholesterol, 5% olive oil and 0.5% cholic acid for 2 weeks in Wistar rats [18, 20]. Our present findings confirm these aforementioned studies showing that isolated hypercholesterolemia induces liver injury. Interestingly, the presence of cholesterol in the diet is necessary for triglyceride accumulation in the liver, as 5% dietary fat alone has no such effect [45]. The mechanisms by which hypercholesterolemia lead to excessive alterations in lipid profile are not clear. Gene regulatory effects of cholesterol are not fully understood yet, however, mechanistic studies showed that the sterol regulatory element-binding protein is a membrane-bound protein which could be cleaved due to hypercholesterolemia, and its intracellular remnant acts as a transcription factor subsequently binding to the sterol regulatory element sequence of DNA [46–48].

In analogy to the effects of isolated hypercholesterolemia on the liver, we presumed that isolated hypercholesterolemia may induce morphological damages in the pancreas as well. There are some reports showing that combined hyperlipidemia has an exacerbating effect in acute pancreatitis [21–25]. However, we could not reveal any vacuolization of acinary cells or an increase in the activity of serum lipase, a pancreas damage-specific marker, induced by isolated hypercholesterolemia. Amylase activity was significantly increased in our study, however, amylase is less specific for pancreas damage and may have extrapancreatic origin. Interestingly high fat diet increases both liver and salivary amylase activity [49, 50]. Pancreatic steatosis has recently been investigated in details. Systemic conditions, e.g. dyslipidemia, obesity may lead to the development of fatty infiltration of the pancreas as well as the liver [23, 24, 27]. Some studies have reported that experimental combined hyperlipidemia causes increased triglyceride accumulation in the pancreas and lipid droplets in acinary cells, i.e. pancreatic steatosis in different animals [23, 27]. In contrast, triglyceride accumulation and histological signs of pancreatic steatosis failed to develop due to isolated hypercholesterolemia in the present study. Therefore, one may speculate that elevation of plasma triglycerides is essential to the development of acute pancreatitis and pancreatic steatosis.

The lack of obesity and the fact that there were no differences in plasma insulin and serum glucose and triglyceride levels in our model suggest that isolated hypercholesterolemia does not lead to massive alterations in the endocrine function of the pancreas. Although our study is limited in this aspect as we have not specifically investigated this issue.

Tissue lipid accumulation is often associated with increased nitro-oxidative stress in various organs, therefore we have also investigated the effect of isolated hypercholesterolemia on nitro-oxidative stress in the liver. Our findings showing elevated hepatic nitro-oxidative stress due to isolated hypercholesterolemia is in line with our own observations made previously in the heart [32, 37, 42].

Since we revealed significant structural alterations macroscopically and marked biochemical changes in the overall lipid content of the liver, we next analyzed the composition of this lipid accumulation by using gas chromatography. Almost all fatty acids we examined showed a significant elevation in the liver in response to isolated hypercholesterolemia. The explanation for the increased overall fatty acid level observed in isolated hypercholesterolemia is not clear and should be investigated in separate studies, since we have not found increased FASN expression in our study. In contrast, amount of some fatty acids, i.e. stearic acid (C18:0) and arachidonic acid (C20:4n-6), showed a significant decrease. These results are very similar to those reported by Muriana et al. in rats fed a diet containing 10% olive oil or primrose oil with or without 1% cholesterol showing no change in stearic acid and a significant decrease in arachidonic acid due to cholesterol supplementation [51]. Among all increased fatty acids the monounsaturated ones (palmitoleic acid (C16:1n-7) and oleic acid (C18:1n-9)) showed the most pronounced alteration in our present study. A feasible explanation for these findings could be an increase in hepatic SCD activity, since SCD convert stearic acid and palmitic acid to oleic acid and palmitoleic acid, respectively. Indeed, SCD1 activity was significantly increased at the transcript level in the liver of hypercholesterolemic rats, however, SCD2 activity failed to increase at the transcript level in hypercholesteremic livers in the present study (Fig. 7).

Palmitoleic acid (C16:1n-7) decreases the activity and expression of LDL-receptors, therefore, elevates the serum concentration of cholesterol [3]; whereas oleic acid (C18:1n-9) and stearic acid (C18:0) act reversely [14]. Oleic acid (C18:1n-9) and stearic acid (C18:0), however, promotes the fatty degeneration, i.e. steatosis, of hepatic cells in different models [52–55]. Furthermore, palmitoleic acid (C16:1n-7) especially, but other fatty acids as well, activate uncoupling protein 2, which may lead to hepatic steatosis [56, 57].

In addition to monounsaturated fatty acids, most of the polyunsaturated fatty acids [most significantly eicosatrienic acid and α-linolenic acid] were also increased in the liver due to isolated hypercholesterolemia (Table 3). Eicosatrienoic acid (C20:3n-3) was shown to be directly converted to leukotrienes [58] which may play an important role in the pathogenesis of hepatic steatosis.

The effects of decreased expression of both FADS1 and FADS2 were clearly visible in our present study as a blockade in the polyunsaturated fatty acid (PUFA) synthesis. The two independent families of PUFAs include namely the n-3 (ω-3, Fig. 8) and n-6 (ω-6, Fig. 9) fatty acids which changed in a similar manner. The amount of the end products (docosahexaenoic acid in the n-3 and arachidonic acid in the n-6 family) did not show any elevation in response to isolated hypercholesterolemia due to decreased expression of FADS1 and FADS2 (i.e. Δ5- and Δ6-desaturases) limiting the synthesis of docosahexaenoic acid and arachidonic acid (Figs. 8 and 9). The late intermediers of both biochemical pathways e.g. formation of tetraenoic, pentaenoic and hexaenoic acids were less pronounced than the very early intermediates. These changes in the PUFA profile in the liver and in the serum can be explained by the substrate accumulation caused by decreased expression of FADSs. n-3 PUFA are able to coordinate an upregulation of lipid oxidation and a downregulation of lipid synthesis, thus n-3 PUFA depletion leads to important metabolic alterations in the murine liver. Moreover, hepatic steatosis can also occur through a mechanism independent of the shift between β-oxidation and lipogenesis [58]. In Pachikian’s work a slight increase in total cholesterol was observed in low n-3 mice confirming the strong relation between n-3 PUFA and cholesterol metabolism [59]. Cellular enrichment of ≥20 PUFAs beyond the rate-limiting FADS2 enzyme are equally effective in preventing atherosclerosis and hepatosteatosis [60].

Nevertheless, the precise physiologic and pathologic role of the hepatic fatty acid alterations observed in our model of isolated hypercholesterolemia need to be further investigated in future studies. Moreover, testing the potential beneficial effects of traditional drugs (e.g. statins), nutraceuticals and functional foods (e.g. resveratrol, plant sterols and fish oil) on isolated hypercholesterolemia induced liver steatosis could be a future direction [61]. Moreover, although isolated hypercholesterolemia did not cause any significant morphological or biochemical alterations indicating steatosis in the pancreas in our study, the unlikely possibility that isolated hypercholesterolemia may still modify pancreatic fatty acid composition, cannot be excluded.

## Conclusions

In summary, isolated hypercholesterolemia is a well-established clinical entity affecting approximately 20% of the hyperlipidemic population. Despite its pronounced relevance, with the exception of this study, investigations have not focused on this topic. The present study revealed that isolated hypercholesterolemia increases the cholesterol and triglyceride content and influences the fatty acid profile of the liver without affecting the pancreas. Altered PUFA synthesis may be explained by the increased hepatic expression of SCD1 and the decreased hepatic expression of FADS1 and FADS2. These findings may indicate that isolated hypercholesterolemia is responsible for the early steps of fat accumulation in the liver. Therefore, fatty acid targeted therapies with pharmacological tools could presumably form the basis of a new strategy in treating or preventing steatosis induced by isolated hypercholesterolemia.

## Abbreviations

FADS1: fatty acid desaturase 1 (Δ5-desaturase)
FADS2: fatty acid desaturase 2 (Δ6-desaturase)
FASN: fatty acid synthase
HPRT1: hypoxanthine-guanine phosphoribosyltransferase-1
PPIA: peptidylprolyl isomerase A
qPCR: quantitative polymerase chain reaction
SCD1: stearoyl-coenzyme A desaturase 1 (Δ9-desaturase)
SCD2: stearoyl-coenzyme A desaturase 2 (Δ9-desaturase)
